# Effects of açai on oxidative stress, ER stress, and inflammation-related parameters in mice with high fat diet-fed induced NAFLD

**DOI:** 10.1038/s41598-019-44563-y

**Published:** 2019-05-30

**Authors:** Mayara Medeiros de Freitas Carvalho, Nara Nunes Lage, Alice Helena de Souza Paulino, Renata Rebeca Pereira, Letícia Trindade de Almeida, Tales Fernando da Silva, Cíntia Lopes de Brito Magalhães, Wanderson Geraldo de Lima, Marcelo Eustáquio Silva, Maria Lucia Pedrosa, Joyce Ferreira da Costa Guerra

**Affiliations:** 1 Research Center in Biological Sciences; Postgraduate Program in Biological Sciences, Federal University of Ouro Preto, Ouro Preto, MG Brazil; 2 Department of Biological Sciences, Federal University of Ouro Preto, Ouro Preto, MG Brazil; 3 Department of Foods, Federal University of Ouro Preto, Ouro Preto, Minas Gerais Brazil; 4 Institute of Biotechnology, Federal University of Uberlândia, Patos de Minas Campus, Patos de Minas, MG Brazil

**Keywords:** Lipids, Dyslipidaemias

## Abstract

Non-alcoholic fatty liver disease (NAFLD), the most predominant liver disease worldwide, is a progressive condition that encompasses a spectrum of disorders ranging from steatosis to steatohepatitis, and, ultimately, cirrhosis and hepatocellular carcinoma. Although the underlying mechanism is complex and multifactorial, several intracellular events leading to its progression have been identified, including oxidative stress, inflammation, mitochondrial dysfunction, apoptosis, and altered endoplasmic reticulum (ER) homeostasis. Phenolic compounds, such as those present in açai (*Euterpe oleracea* Mart.), are considered promising therapeutic agents due to their possible beneficial effects on the prevention and treatment of NAFLD. We tested *in vitro* effects of aqueous açai extract (AAE) in HepG2 cells and its influence on oxidative stress, endoplasmic reticulum stress, and inflammation in a murine model of high fat diet-induced NAFLD. *In vitro* AAE exhibited high antioxidant capacity, high potential to inhibit reactive oxygen species production, and no cytotoxicity. *In vivo*, AAE administration (3 g/kg) for six weeks attenuated liver damage (alanine aminotransferase levels), inflammatory process (number of inflammatory cells and serum TNFα), and oxidative stress, through the reduction of lipid peroxidation and carbonylation of proteins determined by OxyBlot and modulation of the antioxidant enzymes: glutathione reductase, SOD and catalase. No change was observed in collagen content indicating an absence of fibrosis, stress-related genes in RE, and protein expression of caspase-3, a marker of apoptosis. With these results, we provide evidence that açai exhibits hepatoprotective effects and may prevent the progression of liver damage related to NAFLD by targeting pathways involved in its progression.

## Introduction

Non-alcoholic fatty liver disease (NAFLD), which comprises a broad spectrum of conditions characterized by the accumulation of triacylglycerols in hepatocytes, leads to steatosis, steatohepatitis, and can progress to cirrhosis and hepatocellular carcinoma^1^. Thus, the increasing prevalence of NAFLD worldwide is of considerable clinical concern. The abnormal accumulation of triacylglycerols in hepatocytes, i.e., steatosis, is considered the ‘*first hit*’ and most prominent stage in NAFLD pathogenesis. This increases the likelihood of occurrence of advanced stages of the disease, such as inflammation, oxidative stress, mitochondrial dysfunction, endoplasmic reticulum (ER) stress, and apoptosis, which comprise ‘*multiple hits*’ that are responsible for the evolution of the disease^2,3^.

Oxidative stress represents the disruption/dysregulation of signalling and redox balance caused by the increase of reactive oxygen species (ROS), which induce oxidative damage in lipids and proteins^4,5^, and/or a reduction in the antioxidant defence system of an organism, which can include enzymatic and non-enzymatic antioxidant systems. ROS are neutralized by enzymes of the antioxidant defences system and glutathione system (GSH)-related enzymes, besides the superoxide dismutase (SOD), and catalase (CAT) convert ROS to stable compounds, whereas non-enzymatic antioxidant compounds can be obtained through the diet^6^.

ER stress plays an important role in the development and progression of steatosis and NAFLD progression^2^. ER homeostasis occurs via the activation of unfolded protein response to molecular markers including C/EBP homologous protein (CHOP), spliced X-box-binding protein-1 (sXBP1), and activating transcription factor 4 (ATF4), which function to ameliorate the stressed state of the ER^7^. However, the prolonged stimuli of ER stress trigger the inflammatory response through the activation of nuclear transcription factor kappa B (NF-κB) and the production of tumour necrosis factor alpha (TNF-α), thereby initiating apoptosis owing to the continuous expression of CHOP, which suppresses anti-apoptotic factors^8^.

Currently NAFLD has no specific pharmacological treatment established. The bioactive compounds and extracts rich in phenolic compounds have been identified as promising agents in the prevention and/or treatment of NAFLD^9^, mainly those of the anthocyanin class^10^. Such effects are likely due to the broad spectrum of biochemical and pharmacological actions traditionally attributed to their antioxidant capacity in various and complementary mechanisms^11^.

*Euterpe oleracea* Mart., popularly known as açai, constitutes a palm tree fruit usually found in the Brazilian Amazonas and Pará states that has recently attracted considerable attention as a healthy food. Açai contains high amounts of phenolic compounds, such as polyphenols and anthocyanins, which exhibit a beneficial antioxidant activity^12^. In preclinical studies, açai prevented metabolic syndrome^13^, obesity-related adiposity, and hepatic steatosis^14^. Additionally, the use of açai in animal models prevented the progression of oxidative stress biomarkers^15^ along with exhibiting hypocholesterolaemic^16^ and hepatoprotective^17,18^ effects when administered in conjunction with a hyperlipidaemic diet.

The current study was designed to elucidate the contribution of previously unexplored factors of açai administration in an established murine model of NAFLD induced by a high-fat diet. Our results showed that açai treatment improved liver damage parameters, antioxidant status, and reduced inflammation. In this model other parameters related with the NAFLD progression was not change such as, fibrosis, stress ER-related genes, and caspase-3 (CASP-3) protein levels.

## Results

### Aqueous açai extract (AAE) functions as an antioxidant by effectively inhibiting ROS, without exhibiting cytotoxicity

**Figure 1:**
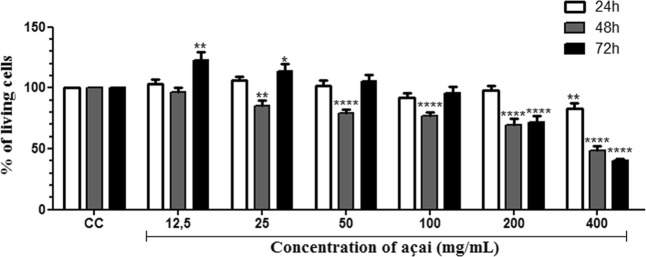
Effects of AAE on cell viability. HepG2 cells were incubated for 24, 48, and 72 h with indicated concentrations of sterile AAE using the MTT method. The assay was performed in octuplicate, using untreated cells as a control (CC), to which 100% cell viability was attributed. *p < 0.05, **p < 0.01, ***p < 0.001, and ****p < 0.0001, ANOVA followed by the Bonferroni test.

The açai pulp showed an excellent antioxidant effect against peroxyl radicals, with substantial total oxygen radical absorbance capacity (ORAC) of 36.608 µmol Trolox equivalents (TE)/mL. AAE in different concentrations did not present cytotoxicity towards HepG2 human liver carcinoma cells during 24 h. At 48 and 72 h, at the concentrations of 200 and 400 mg/mL, toxicity was observed with a dose-response profile. Thus, the CC50 was calculated considering the concentrations of 100 to 400 mg/mL, yielding values of 386.63 and 350.21 μg/mL for 48 and 72 h respectively (Fig. 1).

**Figure 2:**
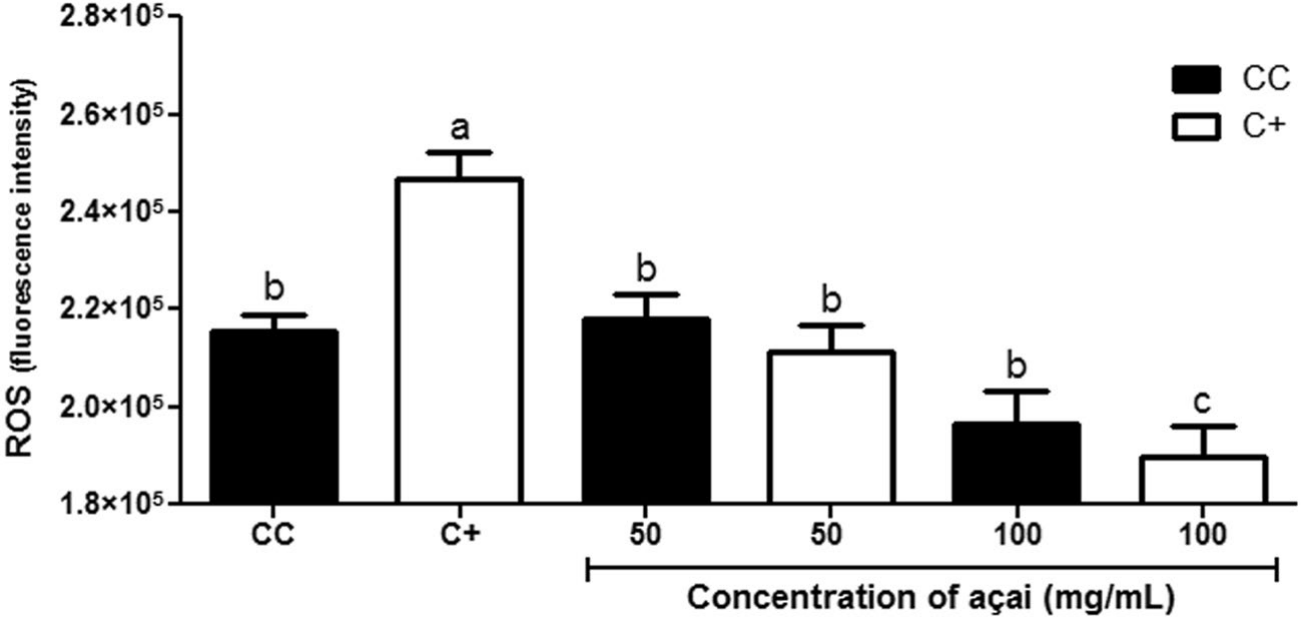
AAE inhibits the formation of EROS induced by tert-butyl hydroperoxide (TBHP) in HepG2 cells and different concentrations of AAE. The assay was performed in octuplicate, using untreated cells (CC) and cells treated with TBHP (C+), as controls. Significantly different values are marked with different superscript letters.

The ROS inhibition assay showed that cells treated with 50 mg/mL of açai demonstrated the same behaviour as the control cells. In the presence of the highest concentration of açai (100 mg/mL), lower levels of ROS were observed in tert-butyl hydroperoxide (TBHP)-treated cells, when compared to the untreated and the açai-treated (100 mg/mL) controls (Fig. 2).

### AAE decreases steatosis, inflammatory cells number, and liver weight, along with alanine aminotransferase (ALT) and TNFα levels in serum

**Figure 3:**
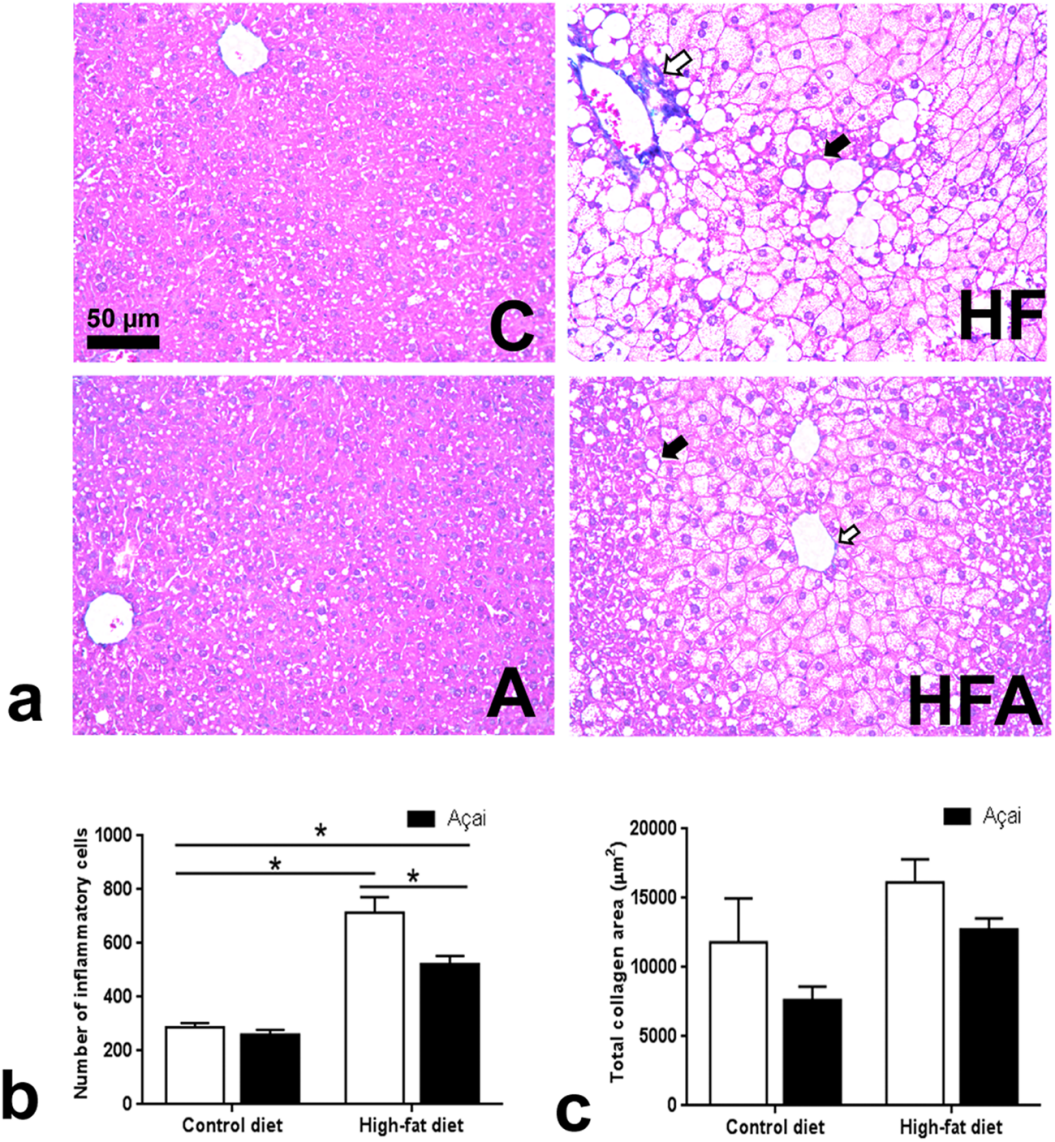
AAE reduces steatosis and inflammation. (**a**) Representative pictures of liver tissue stained with Masson’s trichrome from various experimental groups. White arrows indicate the presence of inflammatory infiltrate and black arrows point to macrovesicular steatosis. C: Control, A: AAE, HF: High-fat, HFA: High-fat + AAE. Bar = 50 μm; 20× magnification. (**b**) Number of inflammatory cells. (**c**) Total collagen area. In (**b**,**c**), data are represented as the means ± standard deviation. *p < 0.05, **p < 0.01, ***p < 0.001, ****p < 0.0001, ANOVA followed by the Bonferroni test.

In this study, histological analyses of the liver were performed to evaluate the extent of inflammation and collagen content. These analyses were represented in a histological panel for each group (Fig. 3a). Our results indicated that the high fat (HF) diet was effective in increasing inflammation, as evidenced by increased levels of TNF-α in the serum and the number of inflammatory cells in the liver (Table 1 and Fig. 3b). The serum levels of TNFα showed a positive correlation with hepatic fat content (r = 0.2421; p = 0.0076), number of inflammatory cells (r = 0.1625; p = 0.0301), and lipid peroxidation in the liver (r = 0.1771; p = 0.0206). Conversely, the administration of açai prevented the increase of TNF-α in HFA mice. However, no differences were found regarding the collagen area (Fig. 3c).

**Table 1:** Effect of AAE on food intake, weight gain, adiposity index, hepatic fat content, liver weight, and AST, ALT, and TNF-α in the serum of mice feed with HF diet.

Parameter	Experimental groups			
	C	A	HF	HFA
Food intake (g/week)	26.46 ± 0.26^a^	24.46 ± 0.28^a^	19.98 ± 0.08^b^	21.48 ± 0.13^b^
Weight gain (g)	26.23 ± 1.21^b^	21.84 ± 1.63^b^	37.48 ± 2.78^a^	35.50 ± 2.07^a^
Adiposity index (%)	6.25 ± 0.70^b^	4.55 ± 0.57^b^	8.90 ± 0.24^a^	7.25 ± 0.56^a^
Hepatic fat content (%)	10.98 ± 1.53^c^	7.78 ± 1.23^c^	30.98 ± 2.01^a^	18.03 ± 1.54^b^
Liver weight (g)	2.17 ± 0.13^b^	1.91 ± 0.04^b^	4.18 ± 0.35^a^	3.15 ± 0.35^b^
***Serum***				
AST (IU)	41.54 ± 2.19	44.27 ± 2.20	47.84 ± 6.53	42.74 ± 3.90
ALT (IU)	10.52 ± 1.49^b^	11.42 ± 1.97^b^	20.89 ± 2.58^a^	11.78 ± 1.43^b^
TNF-α (pg/mL)	14.00 ± 0.43^b^	13.01 ± 0.39^b^	17.74 ± 1.20^a^	14.47 ± 0.50^b^

Values are expressed as the means ± SEM (n = 8). Within a row, significantly different values are marked with different superscript letters.

Assessment of food intake indicated that the administration of the HF diet reduced the amount of food intake in both HF and HFA groups. In addition, we observed that the composition and caloric density of the HF diet increased animal weight gain and adiposity index. In contrast, the administration of açai alone did not alter these parameters. Groups that were fed the HF diet showed increase in liver weight, liver fat percentage, and ALT and TNF-α levels, with these liver changes all being characteristic of steatosis, whereas treatment with açai effectively inhibited the increase of these parameters (Table 1). No effect was detected either for the HF diet or for the açai treatment regarding AST enzyme levels.

### AAE improves antioxidant enzymes and oxidative status

Glutathione (GSH) is considered an important antioxidant defence system in its various (reduced and oxidised (GSSG)) forms. The levels of total GSH in açai (A), HF, and HFA groups increased; however, the same pattern was not observed for GSSG. In the groups that received açai, GSSG values remained similar to those of the control group (Table 2).

**Table 2:** Effect of AAE on antioxidant enzymes in mice feed with HF diet.

Parameter	Experimental groups			
	C	A	HF	HFA
GSH (nmol/mL)	131.00 ± 4.30^b^	161.60 ± 10.01^a^	173.10 ± 8.26^a^	178.90 ± 4.86^a^
GSSG (nmol/mL)	15.53 ± 2.13^b^	18.16 ± 1.82^b^	23.80 ± 1.83^a^	21.01 ± 1.83^b^
GPx (U/g of protein)	2.85 ± 0.09^a^	2.45 ± 0.07^b^	0.41 ± 0.05^c^	0.29 ± 0.03^c^
GR (U/g of protein)	0.16 ± 0.01^b^	0.17 ± 0.01^b^	0.21 ± 0.01^a^	0.19 ± 0.005^ab^
SOD (U/g of protein	0.44 ± 0.05^a^	0.39 ± 0.03^a^	0.28 ± 0.03^b^	0.33 ± 0.02^ab^
CAT (U/g of protein)	108.30 ± 2.23^a^	98.72 ± 3.30^a^	92.61 ± 1.72^b^	109.90 ± 2.30 ^a^

Values are expressed as the means ± SEM (n = 8). Within a row, significantly different values are marked with different superscript letters. GSH: reduced glutathione; GSSG: oxidized glutathione; GPX: glutathione peroxidase; GR: glutathione reductase; SOD: superoxide dismutase; CAT: catalase.

The activity of endogenous antioxidant enzymes was also determined. SOD, CAT, and GPx enzymes showed a reduction in the HF group, whereas the activity of glutathione reductase (GR) in the HF group was higher than that in the untreated control group. Treatment with açai prevented the HF-diet-mediated reductions in SOD and CAT activity and increase in GR activity. However, it did not promote changes in GPx levels (Table 2).

**Figure 4:**
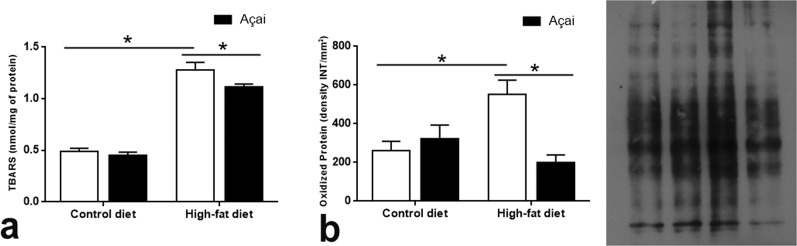
AAE improves oxidative stress. Levels of oxidative stress biomarkers MDA/TBARS (**a**) and oxidized protein (b) in mice. Values are expressed as the means ± SEM (n = 8 and 6, respectively). *p < 0.05, **p < 0.01, ***p < 0.001, ****p < 0.0001, ANOVA followed by the Bonferroni test.

Levels of thiobarbituric acid-reactive substances (TBARS) serve used as an indicator of lipid peroxidation. The HF group showed approximately 2.6 times higher TBARS levels when compared to the C group (p < 0.0001), whereas treatment with açai significantly reduced the levels of these substances (p < 0.05). Similarly, in the HF group an increase in the expression of oxidized protein was observed although the administration of açai prevented the augmentation of this marker, such that the HFA group exhibited levels similar to those of the normal diet-fed controls (Fig. 4).

### AAE and high-fat diet do not induce ER stress

**Figure 5:**
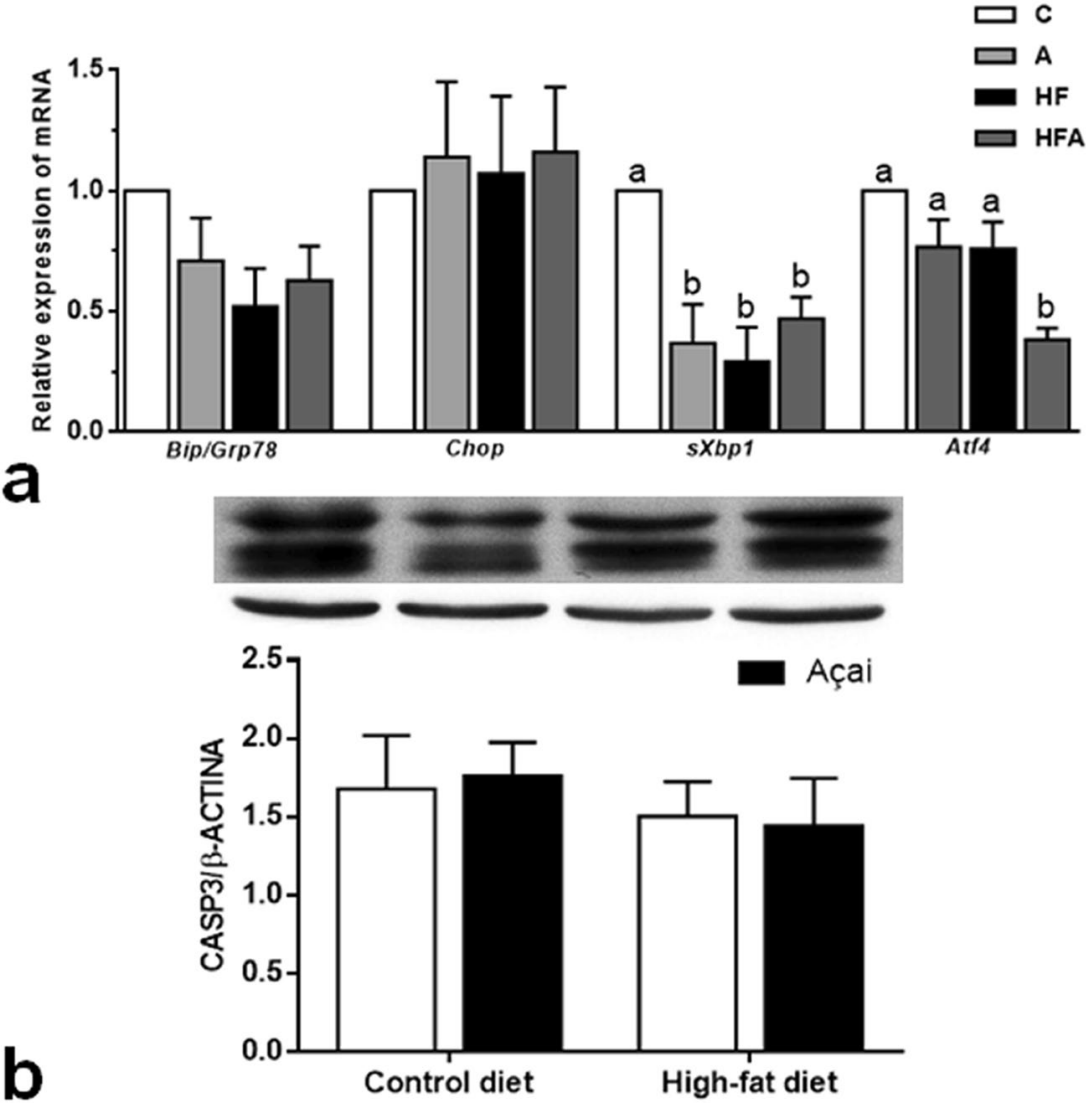
Relative mRNA expression of genes in the liver involved in ER stress pathways and apoptosis in mice. *Bip/Grp78* (chaperone protein); *Atf4*: activity transcription factor 4; *Chop*: C/EBP homologous protein; *sXbp1*: spliced X-box-binding protein-1. Values are expressed as the means ± SEM (n = 6); Significantly different values are marked with different superscript letters.

We analysed the relative mRNA expression of the chaperone BIP/GRP78, a regulator of the unfolded protein response, as well as CHOP, sXBP1, ATF4, along with protein expression of CASP-3, which are important genes for the repression of the ER stress pathway (Fig. 5a). We observed that our experimental model was not effective in inducing ER stress, nor did açai alter those parameters. No difference was observed between groups when the protein expression of CASP-3 was considered (Fig. 5b).

## Discussion

In this study, we examined previously unexplored factors of the protective effects of açai in steatotic mice. We analysed the beneficial effects of açai in factors involved in the progression of NALFD and evidenced that the administration of açai ameliorate the liver damage parameters, inflammation and antioxidant status. In this experimental model other pathways involved did not alters, like collagen content, ER-related stress genes, and CASP-3 production in the experimental groups.

Açai contains several bioactive secondary metabolites, including potential antioxidants, such as flavonoids, mainly anthocyanins and phenolic acids^12^. Numerous studies have described the actions of anthocyanins in the regulation of lipid metabolism and inflammation; however, the doses, in most cases, are impractical for a normal human diet. Therefore, we conducted this investigation using the format (AAE) and dosing of açai that was previously employed by Guerra, *et al*.^17^ to estimate physiological levels of consumption that could be easily ingested with a normal diet.

The ORAC value of AAE was 36.608 µmol TE/mL, which was lower than that previously reported using freeze-dried açai (997 µmol TE/g) or an extract rich in anthocyanins (1800 µmol TE/g) by Schauss, *et al*.^19^ and Hogan, *et al*.^20^, respectively. However, the extract of açai used in the present study exhibited considerably higher ORAC value than those found in common phenolic-rich fruits such as strawberry and blackberry, with ORAC values in the ripe stage of 14.9 and 22.4 µmol TE/g fresh weight, respectively^21^, suggesting that AAE constitutes an exceptional source of natural antioxidants. The response profile of AAE cytotoxicity was similar to that previously demonstrated by Hogan, *et al*.^20^ for an açai, extract rich in anthocyanins, toward another type of cells. Notably, even evaluating higher concentrations of açai over longer exposure periods, we still found that the CC50 values could be considered acceptable.

In addition, AAE exhibited a substantial inhibitory effect on ROS formation in HepG2 cells. At a concentration of 50 mg/mL, açai showed the same behaviour as the control cells, regardless of the presence of the ROS inducer, whereas at 100 mg/mL it was able to further reduce the ROS concentration even in the presence of the ROS inductor. These findings differed from those described by Schauss, *et al*.^19^ for a freeze-dried açai extract, the ROS formation was restored at all açai dilutions tested in human polymorphonuclear cells. We consider that the findings of the present study are particularly relevant because the HepG2 cell line is used to identify toxic compounds in humans as they afford easy manipulation and provide results reproducible in human primary hepatocytes, owing to the metabolic similarity^22^.

Together, our findings demonstrated that AAE exhibits a marked antioxidant activity, in addition to not presenting cytotoxicity yet effectively inhibiting the production of ROS *in vitro*. To facilitate subsequent *in vivo* analyses, the açai, as a natural and safe source, was used, and the dose managed was the same as that used by Guerra, *et al*.^17^ in an animal model capable of exerting a protective effect against NAFLD. In comparison, the average human dose of daily intake for açai would be approximately 200 g for a 70 kg individual, corresponding to approximately 3 g/kg body weight^17^. Notably, the consumption of açai has also previously been shown to have beneficial effects on insulin resistance in overweight individuals^23^. In healthy women, the effects of açai consumption at this dose suggested that it affords protection against atherogenesis and other degenerative diseases related to oxidative stress and dysfunctions in lipid metabolism, improved the antioxidant status by increasing the activity of the enzyme catalase and total antioxidant capacity in polymorphonuclear cells, and decreasing the production of oxygen reactive species^24,25^. Moreover, this dose improved body composition, dietary, clinical, biochemical, and inflammatory parameters in women with obesity^26^.

*In vivo*, our results showed that mice fed the HF diet developed hepatic steatosis and some features related to NAFLD progression. The spectrum of the disease includes oxidative stress, inflammation, activation of liver stellate cell, collagen formation, and consequent fibrosis^5^, our model showed an intense presence of fat droplets, and, in relation to the other markers of progression, we observed inflammation but no collagen deposition in the liver. These alterations are in accordance with the changes in liver weight, liver fat percentage, and serum levels of ALT. Additionally, these changes, which can be caused by HF diets, increased the production pattern and secretion of proinflammatory cytokines, such as TNF-α. Thus, our study complements the findings by Guerra, *et al*.^17^, which showed that this model exhibited an intense accumulation of triglycerides in the liver, lipid deposition, and significant increase in the steatosis score.

We further demonstrated that açai was able to attenuate hepatic steatosis, as evidenced by the reduction in lipid content and based on histological analyses. This effect contributed to preventing the increase of liver weight and serum ALT in AAE treated mice. The açai fruit is known to have hepatoprotective effects in different models and similar results were obtained in other studies^13,17,27^.

AAE also showed anti-inflammatory potential as evidenced by reduced serum TNF-α levels and inflammatory cells in the liver. These results are important to reinforce the anti-inflammatory properties of açai, since it has been well documented that TNF-α serum and hepatic levels are increased in patients with NASH, and it correlates with histological severity of liver damage because there is a strong correlation and direct contribution between necro-inflammatory activity and fibrosis in NAFLD progression^28^.

The inflammatory process has been associated with lipid peroxidation as described in the pathogenesis of NAFLD and is related to the availability of fatty acids. Possible alterations in the oxidant/antioxidant balance correlate with the severity of the disease and are involved in its pathogenesis^29,30^. In the present study, such alterations were evaluated using oxidative stress biomarkers, which indicated an increase in oxidative stress in the mice fed the HF diet as evidenced by the higher concentration of TBARS and the expression of oxidized proteins. Moreover, some studies have demonstrated enhanced lipid peroxidation in situations of NAFLD^31,32^ as well as oxidized proteins^32^, with the severity of this oxidative stress being significantly attenuated in the presence of açai. In the present study, we further demonstrated that there is an important relationship between the biomarker of oxidative stress and a proinflammatory cytokine, and also with the lipid content in the liver, demonstrated by the positive correlation between them.

Liver changes may amplify the effects of oxidative stress, where, in the depletion of enzymes of the antioxidant defence system, may occur because of the increase of reactive species in the NAFLD^33^, thereby compromising the non-enzymatic and enzymatic antioxidant defences^32^. In our study, changes in antioxidant defences were confirmed by the lower activity of GPx, SOD, and CAT enzymes. The addition of açai resulted in an improvement of the redox balance, consequent to the improvement of the biomarkers of oxidative stress, in addition to reversing the changes of the enzymes CAT and SOD. However, it is important to note that the presence of phytochemical compounds, especially polyphenols, has been associated with the effects of açai as an antioxidant via both direct^19^ and indirect effects^15,27,34^ in different experimental models.

In the same context, the changes of GSH levels mediated by açai in the A and HFA groups might be explained by increased transcription of γ-glutamyl cysteine synthetase, a limiting enzyme in the synthesis of GSH, as demonstrated in previous studies^34,35^. These results reinforce the antioxidant role of açai and support that the reduction in oxidative stress is important in the protective effect of the fruit in NAFLD. In accordance with this, Qu, *et al*.^36^ showed that the effect of açai in reducing hepatic injury induced by chronic alcohol consumption in rats derived from increased GSH content and SOD activity.

A steatotic liver is more susceptible to circulating fatty acids and their accumulation can induce ER stress and ROS formation, promoting hepatic injury and, in some cases, apoptosis^37^. Thus, the restoration of homeostasis in ER may constitute a possible mechanism that could explain the hepatoprotective effects of açai. To assess whether changes occurred in ER homeostasis in our HF-diet model, we evaluated three molecular markers of the unfolded protein response. Notably, mRNA expression levels of ATF4 and sXBP1 were low in all groups as compared to those in the control; moreover, the presence of açai did not alter these markers. Expression of BIP/GRP78 and CHOP also did not differ in the experimental groups. Consequently, apoptosis pathways did not appear to be activated, suggested by the equivalent quantification of the CASP-3 protein among groups.

In another high-fat diet model, the treatment with resveratrol (200 mg/kg.bw) for 18 weeks yielded a significant reduction in body weight gain, serum levels of triacylglycerols, total cholesterol, and LDL-cholesterol, as well as liver triacylglycerols and total cholesterol, together with a reduction of the accumulation of intracellular lipid droplets^38^. Models of high-fat diet extending beyond 16 and 18 weeks revealed augmented ER markers in hepatocytes, whereas treatment with resveratrol restored these markers as evidenced by BIP/GRP78, ATF4, and CHOP levels^38,39^. In the present study, it is possible that the time (12 weeks) afforded to the experimental model was insufficient to induce ER stress/apoptosis. However, the therapeutic potential of açai was similar to that of resveratrol in improving steatosis-related parameters, even at a lower dose and shorter treatment time.

Overall, these results suggested that the hepatoprotective role of açai in NAFLD is partly mediated by the modulation of oxidant/antioxidant balance, and inflammatory mediators, key factors related to the disease progression.

## Conclusion

Our data showed a remarkable antioxidant activity *in vitro* of AAE and efficacy in inhibiting ROS production in HepG2 cells with no cytotoxicity. The intake of açai in physiological doses led to an improved response to oxidative stress and modulates proinflammatory related markers, which are associated with a protective role against NAFLD features *in vivo*. These findings indicate the beneficial effects of açai against liver damage with a better understanding of the underlying mechanisms and also provide further evidence of its potential as a nutritional therapy.

## Materials and Methods

### Açai pulp preparation and *in vitro* analyses

A single lot of pasteurized frozen açai pulp without colorants or preservatives was obtained from Icefruit Comércio de Alimentos Ltda. (Tatuí, Sao Paulo, Brazil) and stored at −20 °C. The pulp was thawed, filtered (Whatman n°.1 filter paper, Maidstone, England), and the filtrate (aqueous açai extract; AAE) used in subsequent experiments. Açai pulp proximate composition and phytochemicals content were previously described^17^.

AAE antioxidant capacity was determined using the ORAC method, adapted from other published methodologies^40–42^. Briefly, the peroxyl radical, generated by the reaction of 2,2′-azobis (2-amidinopropane) (AAPH) with atmospheric oxygen, reacts with a fluorescent indicator to form a non-fluorescent product that can be measured by the spectrophotometric decay of the fluorescence in the mixture. Fluorescein (78 nM) was used as a fluorescent probe, the AAPH (dihydrochloride) was used at 221 mM, and 6-hydroxy-2,5,7,8-tetramethylchroman-2-carboxylic acid (Trolox) was used for the calibration curve (at 10, 20, 30, and 40 μM). Diluted sample, phosphate buffered saline, or standard (50 μL) was added to an opaque white 96 well plate in triplicate; to which 50 μL of fluorescein was added and incubated for 15 min at 37 °C. Then, 25 μL of AAPH radical was added and the fluorescence was read at 485 nm (excitation) and 535 nm (emission) every min for 1 h in a VICTOR™ X3 plate reader (PerkinElmer). The ORAC values were calculated using the regression equation between the concentration of Trolox and the net area under the curve and are expressed as equivalents of μM of Trolox per litre.

### Cell viability and ROS production in HepG2 cells

AAE cytotoxicity was evaluated by the 3-(4,5-dimethylthiazol-2-yl)-2,5-diphenyltetrazolium bromide (MTT) assay^43^. The HepG2 cell line, obtained from the Laboratory of Biology and Technology of Microorganism from Federal University of Ouro Preto (UFOP), were seeded in 96 well microplates (2 × 10^4^ cells/well) with 100 μL/well Dulbecco’s modified Eagle’s medium-high glucose (DMEM-HG, Cultilab, Campinas, Brazil) supplemented 5% foetal bovine serum (Cultilab), penicillin/streptomycin (200 U/mL, Sigma-Aldrich, St. Louis, MO, USA), and fungizone (2.5 μg/mL, Sigma-Aldrich), and incubated for 24 h at 37 °C in a humidified incubator with 5% CO_2_. The culture medium was subsequently withdrawn, sterile AAE diluted in DMEM-HG medium at different concentrations (0, 12.5, 25, 50, 100, 200, and 400 mg/mL) was added, then further incubation was performed for 24, 48, and 72 h. Results of MTT analysis, described in detail in Supplementary Methods, indicated the extract concentration able to reduce cell growth by 50% (CC50).

The detection of intracellular ROS production was performed on HepG2 cells using the Image-iT™ LIVE Green Reactive Oxygen Species Kit (Invitrogen®, Carlsbad, CA, USA), which allows the detection of intracellular ROS by fluorescence using a fluorogenic marker (5-or-6)-carboxy-2′,7′dichlorodihydro fluorescein diacetate (carboxy-H_2_DCFDA), with adaptations of a published method^44^.

### Experimental design

Male Swiss mice kindly supplied by the Bioterium of Experimental Nutrition Laboratory, School of Nutrition, Federal University of Ouro Preto (UFOP), MG, Brazil, were used and the procedures were carried out in accordance with the approved guidelines and by the Ethics Committee in Animal Research of UFOP (Protocol N°. 2017/25). They were housed in polypropylene cages with four animals per cage and maintained in a controlled temperature (22 ± 2 °C), light (12 h light/dark cycles), and humidity environment (55%), with food and filtered water provided *ad libitum*.

Thirty-two mice, approximately 30 days old and weighing 25 g were divided into two experimental groups of 16 animals each. The control group (C) received a standard AIN-93M diet^45^. The high-fat diet group (HF) received a high-fat diet (32% lard, 1% cholesterol). Diet composition is described in Table 3. After six weeks, the groups were subdivided into groups C and A (standard diet), and HF and HFA (HF diet). The A and HFA groups were treated with açai, administered as a single daily dose (3 g/kg) for six weeks via gavage during the light phase, whereas C and HF groups received an equal volume of distilled water, as previously described in Guerra, *et al*.^17^.

**Table 3:** Composition of the experimental diets (g/kg diet).

Ingredient	Diet	
	AIN-93M standard diet	High-fat diet
Casein	140.0	190.0
Cornstarch	467.5	87.5
Sucrose	100	100
Maltodextrin	155	155
Soybean oil	40.0	40.0
Lard	—	320
Cholesterol	—	10
Choline	2.5	2.5
Mineral mix^a^	35.0	35.0
Vitamin mix^b^	10.0	10.0
Cellulose	50.0	50.0
Carbohydrate (% energy)^c^	76	26
Protein (% energy)^c^	14	14
Fat (% energy)^c^	10	60

^a^Mineral and ^b^vitamin mixture as recommended by the AIN-93M rodent diet. ^c^Conversion factors: protein, 4 kcal/g; fat, 9 kcal/g; sugars, 4 kcal/g.

After 12 weeks in total, mice were fasted for 12 h, anesthetized with isofluorane, and euthanized by total blood collection from adjacent vessels of the brachial plexus. Blood was collected in polypropylene tubes and centrifuged at 3000 *g* for 15 min. Subsequently, serum was removed and stored at −80 °C. Additionally, liver, epididymal, mesenteric, and retroperitoneal white adipose tissue was collected, then washed in saline, dried, and weighed. From the liver, the small hepatic lobe was stored in buffered formalin for histopathological analysis and the remainder of this organ was rapidly submerged in liquid nitrogen, then immediately stored at −80 °C for subsequent analysis. The adiposity index was determined by the sum of the weights of white adipose tissues divided by bodyweight ×100 and expressed as adiposity percent^46^.

### Serum biochemical analysis

Serum concentrations of active ALT (Cat. N° 53), and aspartate aminotransferase (AST) (Cat. N° 52) were determined enzymatically using commercial kits (Labtest, Lagoa Santa, Brazil). Serum TNF-α levels were quantified using commercial enzyme-linked immunosorbent assays (Mouse TNFα ELISA Kit (RAB0477-1KT; Millipore, Billerica, MA, USA).

### Antioxidant profile in liver tissue

SOD was assayed using an SOD kit (706002; Cayman Chemical Company, Ann Arbor, MI, USA), according to manufacturer instruction. CAT activity was determined as described by Aebi^47^. Briefly, we used 100 mg hepatic tissue, the total protein concentration in the samples was determined using the method of Lowry, *et al*.^48^, and the results were expressed as activity per mg protein, in which one unit of CAT is equivalent to the hydrolysis of 1 μmol of H_2_O_2_ per min.

Total GSH content was determined using a kinetic assay by Griffith^49^. Briefly, a 100-mg aliquot of hepatic tissue was homogenized with 1 mL of 5% sulphosalicylic acid and centrifuged at 10,000 *g* (10 min, 4 °C). Supernatant (10 μL) was added to a microplate, 150 μL working mixture (95 mM phosphate buffer (pH 7.0), 0.95 mM ethylenediaminetetracetic acid (EDTA), 48 μM nicotinamide dinucleotide phosphate and adenine (NADPH), 0.031 mg/mL 5,5′-dithiobis(2-nitrobenzoic acid), 0.115 units/mL glutathione reductase, and 0.24% sulphosalicylic acid) was added, incubated for 5 min, then 50 μL NADPH (0.16 mg/mL) was added and samples absorbance was read every 1 min for 5 min in total. For GSSG, 2 μL of 2-vinylpyridine was added to 100 μL hepatic homogenate, the pH adjusted to between 6 and 7 using triethanolamine, followed by 1 h incubation at ambient temperature, then the derivative samples were assayed as described above.

GPx activity was determined according to a method by Paglia and Valentine^50^ with minor modifications. Hepatic tissue (100 mg) was homogenized in 1 mL buffer (50 mM Tris-HCl, pH 7.0; assay buffer). After centrifugation (10,000 *g*, 15 min, 4 °C), 100 μL assay buffer plus 10 μL supernatant and 80 μL of the composite mix containing 0.25 mM NADPH, 2.1 mM GSH, 0.5 U/mL glutathione reductase, and 1 mM sodium azide were added to a microplate. The reaction was started by the addition of 10 μL H_2_O_2_ (0.2 mM), and NADPH decomposition was monitored at 340 nm, via six readings at 10 s intervals.

GR enzymatic activity was determined according to a method by CARLBERG and MANNERVIK^51^. One unit of GR is defined as the amount of enzyme that causes oxidation of 1 μmol of NADPH per min at 25 °C. The specific activity was expressed in units per mg protein, for GPx and GR, by 11 readings at 10 s intervals.

### Determination of hepatic lipid levels

Hepatic lipids were extracted from liver tissue using a chloroform/methanol solution (2:1, v/v), as described by Folch, *et al*.^52^. The total lipid content in the liver was quantified gravimetrically by solvent evaporation.

### Liver lipid peroxidation

Lipid peroxidation was assessed using the TBARS assay by Buege and Aust^53^. Total protein concentration in the samples was determined according to Lowry, *et al*.^48^. Results are expressed as nmol of malondialdehyde/mg protein.

### Histological analysis

Liver tissue fragments were fixed in 10% buffered formalin for 72 h, dehydrated, cleared, and embedded in paraffin. Tissue sections (4 𝜇m) were cut using a microtome (Leica, Wetzlar, Germany), mounted on microscope slides, stained with Masson’s trichrome, and photographed at 400× magnification (Leica Application Suite) using a Leica DM5000 microscope coupled to a digital camera. Fibrosis and inflammation quantification were assessed by evaluation of the total tissue area (1.5 × 10^6^ μm^2^) using 15 images of randomly-selected fields of tissue sections, per animal, obtained using Leica QWin software.

### Real-time quantitative reverse transcription polymerase chain reaction (*q*RT-PCR) assay of ER stress in the liver

Total RNA was isolated from liver using the RNAgents Total RNA Isolation System (Z3105; Promega, Madison, WI, USA) according to manufacturer instruction. cDNA was synthesized from 2 μg total RNA with random primers using the High-Capacity cDNA Reverse Transcription Kit (#4368814; Applied Biosystems, Foster City, CA, USA) following manufacturer recommendation. qPCR was performed using the Power SYBR® Green PCR Master Mix reagent (#4367659; Applied Biosystems) according to manufacturer recommendation, using the Applied Biosystems® 7500 Real-Time PCR System. Primers are listed in the Supplementary Methods.

### Western blot analysis

Liver tissue was homogenized in lysis buffer (100 mM Tris-HCl pH 8, 1% Triton X100, 20% glycerol, 0.2 mM EDTA) containing protease inhibitor cocktail (P8340, Sigma). The homogenate was centrifuged (10,000 *g*, 15 min, 4 °C) and supernatants were collected. Total protein concentration was determined using the assay by Lowry, *et al*.^48^.

CASP-3 levels were measured using western blotting and an anti-CASP3 antibody (1:3000, Caspase-3 8G10 Rabbit mAb #9665, Cell Signaling Technology) and anti-rabbit IgG coupled to horseradish peroxidase (1:5000, anti-rabbit IgG-HRP: sc-2301; Santa Cruz Biotechnology, Dallas, TX, USA). To detect β-actin, a primary mouse monoclonal anti-β-Actin antibody (1:2000, A2228; Sigma-Aldrich) and anti-mouse IgG (1:5000, A4416; Sigma-Aldrich) were used.

### Detection of protein carbonyls by oxyblot procedure

Protein samples were derivatised in a solution containing 10 mM 2,4-dinitrophenylhydrazine-10% trifluoracetic acid, as described in Levine, *et al*.^54^. The reaction was stopped immediately after 20 min of incubation by neutralization with 2 M Tris base and 30% glycerol, then separated by 15% SDS-PAGE with 5 µg of protein load per track, obtaining two identical gels.

The first gel was stained with Coomassie Brilliant Blue R250 (27816, Sigma); the second was transferred electrophoretically to a nitrocellulose membrane, and processed for western blotting using a rabbit anti-2,4-dinitrophenol antibody (1:2000, D9656; Sigma) that had been incubated with anti-rabbit IgG-HRP (1:5000). ECL detection and densitometry were performed as described above.

Additional materials and methods are available as supplementary materials at the Science website.

### Statistical analysis

Data normality was tested using the Kolmogorov–Smirnov test. Parametric data were analysed by one-way analysis of variance (ANOVA) followed by the Bonferroni *post hoc* test and expressed as the mean ± standard error of the mean (SEM). Non-parametric data were analysed using the Kruskal–Wallis test and Dunn *post hoc* test. The results are expressed as medians and interquartile ranges. Differences were considered significant for p < 0.05, where * is p < 0.05, ** is p < 0.01, *** is p < 0.001 and **** is p < 0.0001. All analyses were conducted using GraphPad prism version 5.00 software for Windows (San Diego, CA, USA).

## Supplementary information

Supplementary information

Certificate of English editing

## Data Availability

The experimental data used to support the findings of this study are included in the article and readers can access it through the article content. Raw data regarding the findings and any other information can be requested from the corresponding author of the paper via e-mail.
