# Supplementary material for: Effects of açai on oxidative stress, ER stress, and inflammation-related parameters in mice with high fat diet-fed induced NAFLD
Source: Sci Rep. 2019 May 30;9:8107. doi: 10.1038/s41598-019-44563-y (PMC6542795; doi:10.1038/s41598-019-44563-y)
Supplement: Supplementary file 1 — Supplementary information [file 41598_2019_44563_MOESM1_ESM.docx]

**Supplementary information**

**Effects of açai on oxidative stress, ER stress, and inflammation-related parameters in mice with high fat diet-fed induced NAFLD**

Mayara Medeiros de Freitas Carvalho^1^, Nara Nunes Lage^1^, Alice Helena de Souza Paulino^1^, Renata Rebeca Pereira^1,2^, Letícia Trindade de Almeida^1^, Tales Fernando da Silva^1^, Cíntia Lopes de Brito Magalhães^1,2^, Wanderson Geraldo de Lima^1,2^, Marcelo Eustáquio Silva^1,3^; Maria Lucia Pedrosa^1,2,*^ & Joyce Ferreira da Costa Guerra^4^

^1^ Research Center in Biological Sciences, Federal University of Ouro Preto, Ouro Preto, MG, Brazil.

^2^ Department of Biological Sciences, Federal University of Ouro Preto, Ouro Preto, MG, Brazil.

^3^ Department of Foods, Federal University of Ouro Preto, Ouro Preto, Minas Gerais, Brazil.

^4^ Institute of Genetics and Biochemistry, Federal University of Uberlândia, Patos de Minas Campus, Patos de Minas, MG, Brazil.

**Supplementary Methods**

**MTT** **assay for cell viability assessment.** HepG2 cells (2 × 10^4^ cells/well) were cultured in 96 well microplates. After 24 h, sterile AAE at different concentrations was added with further incubation for the indicated time points. Then, the supernatant was removed, 28 μL of MTT (5 mg/mL) added, incubated for 1:30 h with further addition of 128 μL dimethyl sulphoxide in each well, and the absorbance was read at 495 nm. To evaluate the percentage of cell viability, the equation of the straight line was used, in which 100% viability was attributed to the control. With the obtained data, a linear regression was performed between the concentrations of 100–400 mg/mL.

**Detection of intracellular ROS production.** Briefly, assays were performed on white 96 well polystyrene plates with approximately 2.5 × 10^4^ HepG2 cells seeded in each well and allowed to adhere overnight. Sterile AAE (50 and 100 mg/mL) was added and incubated at 37 °C for 24 h; control wells received 100 μL Roswell Park Memorial Institute (RPMI-1640) medium (Sigma-Aldrich), whereas in positive control wells, 200 μM terbuthyl was added for 3 h, followed by 100 μL probe (25 μM). The plate was then incubated in the dark, washed with Hank’s buffered saline solution, and RPMI medium was added. Fluorescence intensity was measured at 485/535 nm (excitation/transmission) using a plate reader, as published method[^1^](#_ENREF_1).

**Determination of CAT activity.** Hepatic tissue was homogenized in 1 mL phosphate buffer (100 mM, pH 7.2), and centrifuged at 10.000 *g* (10 min, 4 °C). As a biological sample, 10 μL supernatant was added to 50 μL phosphate buffer (100 mM, pH 7.2) and 40 μL distilled water and the reaction was started by the addition of 900 μL H_2_O_2_ (10 mM). The results were expressed as activity per mg protein, in which one unit of CAT is equivalent to the hydrolysis of 1 μmol of H_2_O_2_ per min , as published method[^2^](#_ENREF_2) .

**Determination of GR activity.** Hepatic tissue (100 mg) was homogenized in 1 mL assay buffer (100 mM potassium phosphate, 1 mM EDTA, pH 7.5). After centrifugation (10,000 *g*, 15 min., 4 °C), 10 μL supernatant was added to 60 μL assay buffer plus 100 μL GSSG (2 mM) in a microplate. The reaction was started by adding 23 μL of NADPH solution (2 mM) and the activity was measured by the absorbance at 340 nm. One unit of GR is defined as the amount of enzyme that causes oxidation of 1 μmol of NADPH per min at 25 °C. The specific activity was expressed in units per mg protein, for GPx and GR, by 11 readings at 10 s intervals as published method[^3^](#_ENREF_3).

**TBARS assay for liver lipid peroxidation:** Liver tissues were homogenized using Tris-HCl buffer (20 mM) and kept on ice. The homogenate was transferred to tubes, mixed with trichloroacetic acid (28% w/v in 0.25 N HCl), thiobarbituric acid (1% acetic acid, 0.25 N), and butyl hydroxy-toluene (125 mM in ethanol), then agitated by vortex, heated at 95 °C for 15 min, and placed in an ice bath. The absorbance was measured at 535 nm. TBARS concentration was calculated using 1,1,3,3-tetramethoxypropane as a standard as published method[^4^](#_ENREF_4).

**qRT-PCR primers**: Primers used for qPCR were as follows: 18S rRNA: 5′-GTA AGT GCG GGT CAT AAG-3′ (forward), 5′-CCA TCC AAT CGG TAG TAG C-3′ (reverse); *Bip/Grp78*: 5′-ACT GCT GAG GCG TAT TTG GG -3′ (forward), 5′-CTT TGG TTG CTT GTC GCT GG-3′ (reverse); *Atf4*: 5′-GCA GTG TTG CTG TAA CGG ACA-3′ (forward), 5′-TCG CTG TTC AGG AAG CTC ATC-3′ (reverse); *Chop*: 5′-TCT TGA GCC TAA CAC GTC GAT-3′ (forward), 5′-TCC GGC TGT TAT TCT GGC TC-3′ (reverse); and *sXbp1*: 5′-CTG AGT CCG CAG CAG CTG-3′(forward), 5′-GGC AAC AGT GTC AGA GTC CA-3′ (reverse).

**Western blot analysis** **of CASP-3:** CASP-3 levels were measured using western blotting. The samples were prepared with running buffer, separated by 15% sodium dodecyl sulphate polyacrylamide gel electrophoresis with 20 µg protein/per well using a Bio-Rad mini gel apparatus (Hercules, CA, USA), transferred by electrophoresis to a nitrocellulose membrane (#1620115; Bio-Rad), blocked, and incubated overnight with an anti-CASP3 antibody (1:3000, Caspase-3 8G10 Rabbit mAb #9665, Cell Signaling Technology). Next, it was incubated with anti-rabbit IgG coupled to horseradish peroxidase (1:5000, anti-rabbit IgG-HRP: sc-2301; Santa Cruz Biotechnology). Proteins were visualized using an enhanced chemiluminescence (ECL) reagent (Westar Nova 2.0, Cyanagen, Bologna, Italy) and developed using X-ray film (Kodak, Rochester, NY, USA).

After the membrane was treated by stripping buffer (Mild Stripping Buffer, adapted by Abcam, Cambridge, UK), it was re-blocked and incubated with a primary mouse monoclonal anti-β-Actin antibody (1:2000, A2228; Sigma-Aldrich) for 3 h at room temperature. Then, the membrane was incubated with anti-mouse IgG (1:5000, A4416; Sigma-Aldrich) and processed as described above. The final data were normalized by β-actin. Densitometry was analysed using Bio-Rad Quantity One 1-D Analysis Software.

1 Camini, F. C. *et al.* Oxidative stress in Mayaro virus infection. *Virus research* **236**, 1-8 (2017).

2 Aebi, H. in *Methods in enzymology* Vol. 105 121-126 (Elsevier, 1984).

3 Carlberg, I. & Mannervik, B. in *Methods in enzymology* Vol. 113 484-490 (Elsevier, 1985).

4 Buege, J. A. & Aust, S. D. in *Methods in enzymology* Vol. 52 302-310 (Elsevier, 1978).
